# Thinking (Metastasis) outside the (Primary Tumor) Box

**DOI:** 10.3390/cancers15225315

**Published:** 2023-11-07

**Authors:** Zhe Jiang, Young-Jun Ju, Amjad Ali, Philip E. D. Chung, Dong-Yu Wang, Jeff C. Liu, Huiqin Li, Ioulia Vorobieva, Ethel Mwewa, Ronak Ghanbari-Azarnier, Mariusz Shrestha, Yaacov Ben-David, Eldad Zacksenhaus

**Affiliations:** 1 Toronto General Research Institute—University Health Network, 101 College Street, Max Bell Research Centre, Suite 5R406, Toronto, ON M5G 1L7, Canadayju@lakeheadu.ca (Y.-J.J.); amjad_486@yahoo.com (A.A.); dongyu.wang@utoronto.ca (D.-Y.W.); hqli689@gmail.com (H.L.); ethelmwewa@gmail.com (E.M.); ghronak@gmail.com (R.G.-A.); m.shrestha@mail.utoronto.ca (M.S.); 2 Laboratory Medicine & Pathobiology, University of Toronto, Toronto, ON M5S 1A8, Canada; 3 The Donnelly Centre, University of Toronto, Toronto, ON M5S 3E1, Canada; dr.seamonster@gmail.com; 4 State Key Laboratory for Functions and Applications of Medicinal Plants, Guizhou Medical University, Guiyang 550025, China; yaacovbendavid@hotmail.com; 5 The Natural Products Research Center of Guizhou Province, Guiyang 550014, China; 6 Department of Medicine, University of Toronto, Toronto, ON M5S 3H2, Canada

The metastasis of tumor cells into vital organs is a major cause of death from diverse types of malignancies [1,2,3,4]. One model posits that metastasis is driven by the same oncogenic drivers that promote primary lesions [5] (reviewed in [3]). This model is supported by the high concordance between the oncogenic landscapes of metastases versus primary lesions from the same patients [6]. An alternative model asserts that additional oncogenic drivers promote metastasis and are therefore absent or under-represented in the oncogenic landscape of primary tumors. Indeed, multiple reports uncover new oncogenic mutations in metastatic versus primary lesions [7,8,9,10,11,12,13,14,15,16,17]. However, whether such new mutations represent true metastatic drivers or genes that confer drug-resistance is not entirely clear [18,19]. Elucidating this issue is important not only for understanding metastasis but also for crafting new combination drug therapies to prevent metastatic disease at the time of initial tumor diagnosis.

To address this issue, our group has recently conducted a transposon-based (Sleeping Beauty) mutagenesis screen on drug-naïve, retinoblastoma Rb-deficient genetic background [20] and identified oncogenic drivers in the primary (mammary gland) and metastatic (lung) compartments [21]. We used conditional Rb-mutant mice because the RB tumor suppressor is frequently disrupted in breast cancer either genetically via oncogenic alterations such as mutations/deletions/promoter methylation of the gene or functionally through over-phosphorylation of the pRB protein via cyclin-dependent kinases CDK4/6 and CDK2 [22,23,24]. RB loss dysregulates the cell cycle, cell physiology, and metabolism and induces immune-checkpoint inhibitors such as PD-L1 and PVR [22,23,25,26,27,28,29].

Our genetic screen identified oncogenic drivers that promote primary-only mammary tumors, metastasis-only lung lesions, or both primary and metastatic growth (which we termed “shared” oncogenic drivers). Importantly, using DNA sequence data on the transposon integration sites, we were able to establish clonal relationships between metastasis drivers and primary drivers, thus excluding the possibility of unrelated primary mammary and primary lung tumors. The shared oncogenic drivers comprised a major MET-RAS hub (Met, Prlr, Nf1, Map3k3, Stat5b, and Notch1) known to promote tumor growth and cell migration [30,31,32], as well as Jup/Plakoglobin/gamma-catenin, involved in collective cell migration [33]. The metastasis-specific drivers formed three additional hubs: Rho signaling (migration; Srgap2, Cdc42bpa/Mrcka, and Wasf2/Wave2), ubiquitination (e.g., Fbxw4, Ubxn7, Spop, Hectd1, and Ube2d3) and RNA processing (Wdr33, Cwc22, Cdc5l, Prpf6, and Pspc1) [21]. We showed that the expression of these metastatic hub genes correlates with poor prognosis in breast cancer and validated representatives of each hub for promoting hallmarks of metastasis such as cell migration, as well as cell proliferation and/or tumorigenesis.

To ask whether similar organization occurs in breast cancer patients, we calculated the pathway activity of 26 different oncogenic signaling in several independent cohorts of paired primary and metastatic breast lesions [21,34,35,36,37]. We found a parallel organization with subtype-specific shared oncogenic drivers (e.g., RB1-loss, TP53-loss, high MET, MYC, ER) and across subtypes primary-enriched (EGFR, TGFb, and STAT3) and metastasis-enriched (RHO and PI3K) signaling [21]. These results are consistent with previous observations that the EGFR, TGFb, and STAT3 pathways exhibit opposing effects on primary vs. metastatic cancers [38,39,40,41,42,43]. The metastatic-enriched pathways RHOA and PI3K promote cell migration, which is required for the metastatic cascade. PI3K signaling also enhances survival and drug resistance, and it may therefore be enriched in metastases from drug-treated patients. In contrast, RHOA signaling was one of the hubs seen in the transposon mutagenesis of drug-naïve animals and likely represents a genuine metastatic driver. These and other metastasis-enriched or metastasis-specific drivers may be ideal targets for prevention strategies.

We envisage that primary-enriched oncogenic drivers evolve to promote clonal competition and expansion at the local site. Such drivers may not be compatible with, and may even oppose, the metastatic cascade; therefore, their activity is diminished in metastases [1,2,3,4]. For example, a glycolytic shift facilitates the efficient synthesis of macromolecules required for rapid cell duplication [44,45], yet oxidative phosphorylation (OXPHOS) is a hallmark of circulating, metastatic tumor cells [3,23,46,47]. Thus, tumor cells with the ability to metastasize must maintain cell plasticity to enable shifts from one state to another, such as from glycolysis to OXPHOS and vice versa [3]; epithelial-to-mesenchymal-transition (EMT) required for migration but also mesenchymal-to-epithelial transition (MET) required for growth at distal sites [48]; partial EMT or hybrid amoeboid EMT rather than full EMT to enable group migration and effective metastasis [49,50,51]; and cell proliferation, but with the ability to reversibly enter dormancy or diapause-like states, thereby escaping chemotherapy [52]. We postulate that such plasticity is driven by shared oncogenic drivers. Metastasis-enriched oncogenic pathways likely contribute more to metastasis than to primary tumors and are therefore enriched at distal sites. Metastatic-specific drivers may represent mutations or epigenetic alterations that are critical for the metastatic cascade, enforce rapid dissemination, or are induced upon interaction with the tumor microenvironment (TME); hence, they are uniquely detected at the metastatic compartment (Figure 1). Notably, the cancer cell–TME interaction is reciprocal as is evident, for example, from the expression of immune checkpoint modulators on the surface of tumor cells, which suppress immune surveillance, or from the effect of different tumor exosomal integrins on the pre-metastatic niche in different organs [53]. It is important to stress that tumor dissemination into the blood and lymphatic systems is not the rate-limiting step in metastasis, as a vast number of circulating cancer cells disseminate and reach the circulation, especially during tumor excision [54]. Only a small fraction of circulating tumor cells with rare, pre-existing, or acquired alterations are endowed with the ability to form life-threatening macro-metastases at distal organs.

Two major conclusions can be drawn from this study. First, in line with the title of this Editorial, the therapeutic importance of oncogenic drivers that promote primary cancer (for tumors such as breast cancer that are surgically removed) can only be appreciated by understanding their impact on metastasis. Second, targeting shared- and metastasis-enriched or -specific but not primary-enriched drivers represents a rational strategy to prevent metastases at the time of primary tumor detection [21].

Cognizant of the therapeutic importance of targeting metastasis-enriched pathways in conjunction with shared oncogenic alterations, there is an ever-growing interest in deepening our understanding of metastasis and its druggable targets.

The Special Issue “From Progression to Metastasis of Solid Cancer” (https://www.mdpi.com/journal/cancers/special_issues/Progression_Metastasis_cancer, accessed on 7 November 2023) [3] comprises five original articles and eight reviews. Original articles include mechanisms of the adipsin-dependent secretion of hepatocyte growth factor (HGF) at the adipocyte–cancer stem cell interface from the Yohei Shimono group [55]; characterization of slow-cycling glioblastoma cells—Loic P. Deleyrolle [56]; therapeutic effects of the immune checkpoint plus BRAF/MEK inhibitors for BRAFV600-mutant metastatic melanoma—Stephan Grabbe [57]; the impact of osteoblast-specific factor 2 in the progression and metastasis of head and neck cancer—Roland H. Stauber [58]; and finally, the effects of Yoda1, an activator of the mechanosensitive Piezo1 channel, and low-magnitude high-frequency vibration on cell migration and the survival of breast cancer cells—Lidan You [59].

This Special Issue also contains an Editorial summation of metastasis [3] and insightful reviews on the hepatic pre-metastatic niche from the group of Allan Tsung [60]; AXL receptor tyrosine kinase as a potential therapeutic target for cancer progression and metastasis—Jean-François Côté [61]; impact of BRCA1 loss and defective DNA repair on metastasis—Razqallah Hakem [62]; clinical biomarkers for early detection of intracranial metastases—Sunit Das [63]; effect of the breast tumor microenvironment on metastatic dissemination—Yvonne Myal [64]; leptomeningeal metastases in adult solid cancers—Peter M. Siegel [65]; and integrin-linked kinase (ILK) signaling in metastasis—Shoukat Dedhar [66].

*Cancers* has dedicated multiple other past and ongoing Special Issues to metastatic cancer, including “Targeting Bone Metastasis in Cancers” [67]; “High-Risk Localized and Locally Advanced Prostate Cancer” [68]; “Advances and Novel Treatment Options in Metastatic Melanoma” [69]; “Metastasis and Tumor Cell Migration of Solid Tumors” [70]; “Understanding Molecular Regulation of Cancer Progression and Metastasis” (https://www.mdpi.com/journal/cancers/special_issues/Molecular_Regulation_Cancer_Progression_Metastasis, accessed on 7 November 2023); “Genomic Landscape of Breast Cancer: From Primary to Metastasis” (https://www.mdpi.com/journal/cancers/special_issues/genomic_breast, accessed on 7 November 2023); “Targeting Cancer Metastasis” (volume II) (https://www.mdpi.com/journal/cancers/special_issues/TCM, accessed on 7 November 2023); and “New Insights into the Molecular Mechanism of Epithelial Plasticity in Cancer” (https://www.mdpi.com/journal/cancers/special_issues/Mechanism_Epithelial_Plasticity, accessed on 7 November 2023).

This remarkable collection of articles uncovers intrinsic oncogenic drivers and external factors from the tumor microenvironment, including the immune surveillance system that contribute to metastatic progression. Such oncogenic drivers and factors are ideal targets for combination therapies comprising patient-tailored inhibitors for shared oncogenic drivers that promote both primary and metastatic growth, together with inhibitors for metastasis-specific or -enriched drivers. Future challenges include the further exploration of the novel mechanisms of metastasis and interventions for each cancer type and subtype, as well as the systematic evaluation of drug combinations against shared- and metastasis-enriched or specific pathways, initially in pre-clinical animal models and then in clinical settings.

## Figures and Tables

**Figure 1:**
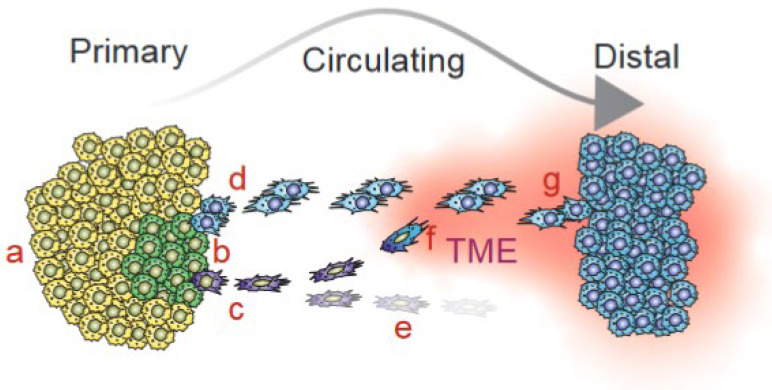
Primary tumor cells (left) evolve via a Darwinian process that selects tumor cell clones (e.g., yellow cells) with the ability to rapidly proliferate, survive, and dominate the primary tumor population locally. However, these cells may lack the plasticity required for metastasis. In contrast, other tumor cells (green), driven by shared oncogenic alterations that promote both primary and metastatic growth with the necessary cell plasticity, are capable of metastatic dissemination. Additional metastasis-enriched or metastatic-specific alterations potentiate tumor cells with a complete (elongated clusters of blue cells) or partial (elongated single purple cells) ability to form macro-metastasis. **a**—primary tumor cells driven by primary-only oncogenic drivers (yellow epithelial cells) dominate the primary tumor landscape but fail to metastasize; **b**—primary tumor cells driven by shared oncogenic drivers (green epithelial cells); **c**—disseminating tumor cells driven by shared oncogenic drivers plus metastasis-enriched or specific drivers with incomplete metastatic potential (elongated purple cells); **d**—disseminating tumor cells driven by shared plus metastasis-enriched/-specific tumor cells that undergo group migration with complete metastatic potential (elongated blue cells); **e**—aborted circulating tumor cells (purple) that fail to survive or form macro-metastases; **f**—circulating tumor cells (purple) that acquired additional mutations or signals from the TME that endowed them with full metastatic potential (elongated purple to blue conversion); **g**—mesenchymal-to-epithelial transition (MET) and the colonization of a distal site (blue epithelial cells). Targeted therapies against 2–3 shared oncogenic drivers or against shared plus metastasis-enriched/-specific drivers but not primary-only drivers offer a rational, neo-adjuvant approach to prevent metastatic cancer upon diagnosis.
